# Healthcare governance during humanitarian responses: a survey of current practice among international humanitarian actors

**DOI:** 10.1186/s13031-021-00355-8

**Published:** 2021-04-10

**Authors:** Prudence Jarrett, Yasin Fozdar, Nada Abdelmagid, Francesco Checchi

**Affiliations:** Faculty of Epidemiology and Population Health, London School of Hygiene and Tropical Medicine, London, UK

**Keywords:** Humanitarian, Crisis, Emergency, Health, Governance, Accountability

## Abstract

**Background:**

Large international humanitarian actors support and directly deliver health services for millions of people in crises annually, and wield considerable power to decide which health services to provide, how and to whom, across a vast spectrum of health areas. Despite decades of reform aiming to improve accountability in the sector, public health practice among humanitarian actors is not heavily scrutinized in either the countries where they are headquartered or those where they provide healthcare. We surveyed current healthcare governance practice among large international humanitarian actors to better understand what organisations are doing to ensure oversight and accountability for health services in humanitarian responses.

**Methods:**

The term ‘healthcare governance’ was defined and categorised into seven domains: implementation of health management information systems (HMIS) and use of resulting data; professional development of health sector staff; audits of health service performance; management of clinical incidents; evidence-based practice; pharmaceutical supply; and beneficiary engagement. Senior health professionals at 32 leading international actors providing humanitarian health services were contacted between July and August 2019 to complete a 109-question online survey about their organisation’s practice in these domains.

**Results:**

Respondents from 13 organisations completed the questionnaire. Healthcare governance practices were undertaken to varying degrees by all organisations but were often driven by donor requirements and external factors rather than improvement of programme performance. Common strengths were the inclusion of governance in organisational policies, high availability of technical guidelines, and close monitoring of pharmaceutical services. Recurring weaknesses were poor beneficiary engagement, inconsistent use of health information for decision making, unsystematic implementation of healthcare audits, inconsistent management of clinical incidents, and lack of training and professional development opportunities.

**Conclusions:**

To our knowledge, this is the first study to describe healthcare governance practice among humanitarian actors. Leading international humanitarian healthcare providers are already implementing many healthcare governance activities; however, these are inconsistently applied and generally not reflective of systematic policies or earmarked organisational resources. There is a need for sector-wide consensus on how the humanitarian sector defines healthcare governance, the domains that constitute it, which actors in the humanitarian system are implicated, and how malpractice should be systematically addressed.

**Supplementary Information:**

The online version contains supplementary material available at 10.1186/s13031-021-00355-8.

## Background

In 2019, 168 million people worldwide required humanitarian assistance [1] to mitigate the effects of armed conflict, natural disasters and political instability. International non-governmental organisations (NGOs), along with United Nations agencies and the Red Cross and Red Crescent movement, receive the vast majority of formal global funding for humanitarian assistance [2] and directly or indirectly support the delivery of critical health services for these populations.

The ‘humanitarian system’ is a network of mostly autonomous actors with varying mandates, funding sources and technical expertise [3]. Actors responsible for providing health services can be seen as forming a sub-system within this broader network. This system is not governed in the conventional sense, with no overarching body to answer to. From the perspective of beneficiaries, multilateral organisations (e.g. the World Health Organization, WHO) are mostly exempt from legal accountability, while other international humanitarian actors are typically neither subject to stringent scrutiny where they are headquartered (e.g. in England and Wales, the Charity Commission does not proactively monitor the quality of NGO services provided abroad) nor in the locations where they provide humanitarian services. While the latter may depend on local legislation and the capacity and intentions of host governments, in practice, most crisis contexts are characterized by weak governance and a breakdown of law and order [3].

Despite this lack of accountability, humanitarian health actors have de facto responsibility. They wield considerable power to decide which health services to provide, how and to whom, across a vast spectrum ranging from epidemic response to antenatal care and trauma surgery. Whether they set up their own health facilities or support pre-existing health services, their role arguably implicates them in ensuring that service delivery attains a minimum acceptable performance. Furthermore, this responsibility may be widely shared – for example, the response to a polio outbreak in opposition-held areas of Syria involved international donors funding international NGOs. The latter, in turn, sub-contracted local NGOs and supported local ex-Ministry of Health clinicians to deliver the response. At the same time, UN agencies provided technical co-ordination and procured vaccines (F Checchi, pers. Comm.).

Signified by the growing field of ‘humanitarian governance’ and a shift towards ethics concerned with the consequences, rather than the duties, of humanitarian aid [4, 5], there have been repeated attempts in recent years to improve the co-ordination and effectiveness of humanitarian response, often after evident systemic failures [4, 6, 7] (Table 1). These may have fostered increasing standardization, professionalization and accountability [18]. For example, the Sphere Project’s [12] minimum standards in humanitarian response, while voluntary and based on imperfect evidence, are widely perceived as useful [19].

**Table 1:** Summary of major reforms and initiatives related to governance and accountability of the humanitarian system

Reform or Initiative	Lead organisation(s)	Description	Year
IASC (Inter-agency Standing Committee)	OCHA (Office for Co-ordination of Humanitarian Affairs)	Humanitarian co-ordination forum for the UN system to ensure coherence of preparedness and response efforts, formulate policy and agree on priorities for strengthened humanitarian action [8].	1992
Code of Conduct	IFRC (International Federation of Red Cross and Red Crescent Societies)	Voluntary code to maintain high standards of independence, effectiveness and impact to which other NGOs are signatories [9].	1994
People In Aid	People In Aid	International network of relief and development agencies committed to improving human resources management through the People in Aid Code of Best Practice [10]. Merged with HAP-I to form CHS Alliance in 2014 [3].	1995
ALNAP (Active Learning Network for Accountability and Performance)	ALNAP	Global network of NGOs, UN agencies, members of the Red Cross and Red Crescent Movement, donors, academics, networks and consultants dedicated to learning how to improve response to humanitarian crises [11].	1997
Humanitarian charter & minimum standards in humanitarian response	Sphere Association	Humanitarian charter is a voluntary commitment of shared principles, rights and obligations for ensuring the welfare of crisis-affected populations. Minimum standards for health, WASH, nutrition, food security and shelter with suggestions for indicators and targets [12].	1997
Humanitarian Accountability Partnership International (HAP-I)	HAP-I	Multi-agency initiative and first body for self-regulation in humanitarian sector. Created the HAP Standard in Accountability and Quality Management. Merged with People in Aid to form CHS Alliance in 2014 [3].	2003
Cluster system	WHO	Co-ordination of humanitarian response at the global and crisis response level to ensure predictable leadership and accountability in all main sectors, strengthen system-wide preparedness and technical capacity in humanitarian emergencies [13].	2005
Transformative agenda	IASC	Set of concrete actions aimed at improving the timeliness and effectiveness of the collective response through stronger leadership, more effective co-ordination structures and improved accountability for performance and to affected people [14].	2011
Core Humanitarian Standard on Quality and Accountability	CHS Alliance (formerly HAP-I and People in Aid), Sphere, Groupe URD, HQAI	Voluntary standard made up of nine commitments with key actions and organisational responsibilities to improve the quality and effectiveness of humanitarian assistance [15].	2014
Humanitarian Quality Assurance Initiative	HQAI	NGO providing services for benchmarking, verification and certification against the Core Humanitarian Standard [16].	2015
Grand Bargain	IASC	Agreement between donor governments, UN agencies and aid organisations to improve the efficiency and effectiveness of international humanitarian aid particularly focussed on transparency, better co-ordination and reform to humanitarian financing [17].	2016

Yet poor decision-making – either through commission or omission - remains common in humanitarian responses [2, 20]. Such malpractice is not well documented, and evaluations of humanitarian action are often of low-quality [21], making it challenging to quantify impacts on beneficiaries. Globally, harmful healthcare practice is estimated to affect one in four outpatients, resulting in 2.6 million preventable deaths annually [22]. Humanitarian responses are highlighted as a particular concern for improving the quality of healthcare provision globally [23].

While clinical malpractice (e.g. unsafe surgical practices, inaccurate diagnosis and prescription) is well recognised, *public health* malpractice (e.g. inappropriate decisions on which health services to provide; abrupt interruption of services; sub-standard service provision) is harder to define, but potentially even more harmful. To the extent that such malpractice occurs in the humanitarian health sector, it wastes resources, damages the reputation of actors with implications for fundraising and recruitment, and causes moral distress for individuals working in the field [24]. Critically, it undermines the primary aim of humanitarian health action to reduce crisis-attributable morbidity and mortality.

In resource-rich settings, a set of deeply institutionalized policies and practices definable broadly as ‘healthcare governance’ has become fundamental to preventing and mitigating malpractice and ensuring public accountability of health services. There is no unifying definition of healthcare governance [25] and most conceptualisations underscore the centrality of national government and long-term strategic planning for health systems making them less applicable to humanitarian health responses [26–28].

The published literature offers few examples of humanitarian actors adopting elements of healthcare governance. Kersten et al. piloted a quality-of-care audit tool in a Médecins Sans Frontières (MSF) project in South Sudan [29], finding deficits across all service areas. Shanks et al. describe experience implementing a voluntary medical error reporting system across MSF projects in 18 countries [30], showing that a substantial proportion of reported errors caused harm (39.1%) or death (23.5%). Both reports focus only on the clinical aspect of healthcare malpractice.

The contribution of the study presented in this paper is therefore as a first step towards a more systematic and sector-wide understanding of healthcare governance in humanitarian crises by surveying current policies and practices relating to healthcare governance in large international humanitarian health NGOs.

## Methods

### Scope and definitions

Given the lack of a definition of healthcare governance appropriate to humanitarian contexts, we expanded the concept of clinical governance first implemented in the United Kingdom National Health Service (NHS) [31] to apply it to the range of healthcare activities conducted by international humanitarian actors. We chose to base our design on the concept of clinical governance for several reasons: first, it can be applied to both state-citizen and NGO-beneficiary relationships; second, it describes responsibilities and processes particular to healthcare delivery and thus provides a framework for asking specific questions about how quality and safety of care are managed in practice; and finally, it is likely to be familiar to health practitioners as well as organisations that provide health services in the humanitarian sector making it a starting point for discussing this topic.

We then defined ‘healthcare governance’ as ‘the set of rules, structures and mechanisms for collective decision-making employed by international humanitarian health actors in the pursuit of providing effective and safe health care’.

**Fig. 1:**
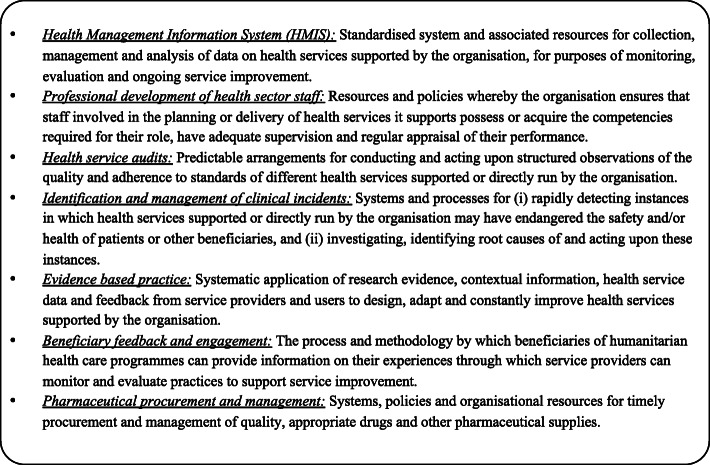
Definitions of the domains of healthcare governance

We further specified seven domains of healthcare governance (based on the ‘seven pillars’ described for the NHS) [32] that we assumed would apply to any modality of humanitarian health action: implementation of health management information systems and use of resulting data; professional development of health sector staff; audits of health service performance; management of clinical incidents; evidence-based practice; pharmaceutical supply; and beneficiary engagement (see Fig. 1 for details). We constrained our survey to the humanitarian health sector, which typically excludes nutrition and water, sanitation and hygiene (WASH) services. Accordingly, we referred to the International Classification of Diseases [33] to define seven broad service areas into which both clinical and public health activites can be categorised: endemic infectious diseases; epidemics; HIV and tuberculosis; maternal, neonatal, reproductive and sexual health; non-communicable diseases; mental health; injuries.

### Survey design

We developed a structured questionnaire (see Additional file 1) based on the seven domains above, consisting of 109 mostly close-ended questions. We invited respondents to provide details or upload relevant organisational documents to illustrate domain-specific policies or practices and add free-text comments at the end of each section.

### Study participants

We selected international NGOs and the Red Cross / Red Crescent (RC) movement, as we wished to investigate policies and practices among actors proximally and operationally involved in health service delivery or support (as opposed to donors or UN specialised agencies that are generally not directly involved in health service provision [34]). As an introductory, rather than a comprehensive, exploration of healthcare governance in the humanitarian health sector, we surveyed the largest international health actors in terms of size and global footprint, and who conceivably have the most significant influence on practice in the sector and health outcomes for crisis-affected populations. Accordingly, we firstly selected all international NGO and RC actors (including national RC societies) that received contributions ≥2.5 million USD during 2018 for health sector projects, according to the Office for the Co-ordination of Humanitarian Affairs (OCHA)‘s Financial Tracking Service (FTS) [35]. We supplemented this list with additional international organisations that were members of the Global Health Cluster as of June 2018 and all operational centres (sections) of MSF (the latter receive mainly private funding and are thus not well captured in the FTS database).

### Survey implementation

Utilising publicly available contact details and our professional network, we invited by email the most senior health technical expert within the organisation (e.g. medical directors at MSF) to complete the survey. Where we could not identify such an individual, we used generic contact information for that organisation to solicit participation. We administered the survey through an online platform (Jisc Online Surveys) [36]. We informed participants that individual organisation responses would be visible to the researchers but anonymised in publications arising from the study.

### Ethics

Participants read an online information sheet and provided online written consent. The study was approved by the London School of Hygiene and Tropical Medicine Ethics Committee (ref. 15704).

## Results

### Profile of responding organisations

Data were collected during August and September 2019. Out of 32 organisations invited, 13 completed the questionnaire with a total of 16 individual responses – referred to as key informants (Table 2); 19 organisations declined due to competing work priorities or did not reply; no refusals were recorded. All 13 organisations were operating in more than one continent. All provided services directly to the affected population, and all but one (12/13, 92.3%) also worked in support of the local health system. They worked at the community (13/13, 100%), primary care (13/13, 100%) and, less commonly, secondary care level (8/13, 61.5%). Just under half (6/13, 46.2%) of the organisations described working across all seven disease areas. The remaining results are reported as individual responses by key informants with variation in the denominator reflecting incomplete data for that question.

**Table 2:** List of participating organisations in alphabetical order with the number of participants from each NGO (HQ = headquarters)

Organisation	Number of respondents	Location
International Federation of the Red Cross	1	HQ
International Rescue Committee (IRC)	1	Country Office
Intersos	1	HQ
Médecins du Monde (MDM) Belgium	1	HQ
Médecins Sans Frontières – Operational Centre Brussels (OCB)	1	HQ
Médecins Sans Frontières – Operational Centre Paris (OCP)	1	HQ
Médecins Sans Frontières – Operational Centre Geneva (OCG)	1	HQ
Médecins Sans Frontières – International Bureau	1	HQ
Medical Teams International	1	HQ
MENTOR Initiative	1	HQ
Mercy Malaysia	1	HQ
Norwegian Red Cross (NRC)	1	HQ
Première Urgence Internationale	1	Country Office
Relief International	1	HQ
Save The Children	1	HQ
World Vision International	1	HQ

All key informants reported that their organisation had a policy on governance and accountability, two-thirds of which (10/15, 66.7%) covered health services. Most reported that there was an executive or team responsible for each of the seven governance domains in the survey. However, a lower proportion reported a formal pathway for escalating issues relating to the domains (Table 3).

**Table 3:** Number (%) of respondents with a designated executive or team and with an established escalation pathway for oversight of each governance area (*N* = 16)

Governance area	Responsible executive or team N (%)	Escalation pathway N (%)
HMIS	13 (81.3)	10 (62.5)
Professional development	12 (75.0)	7 (43.8)
Health audit	12 (75.0)	10 (62.5)
Incident management	10 (62.5)	9 (56.3)
Evidence based practice	12 (75.0)	8 (50)
Beneficiary engagement	13 (81.3)	11 (68.8)
Pharmaceutical services	15 (93.8)	14 (87.5)

### Domains of healthcare governance

#### Health management information system (HMIS)

A majority of organisations implemented a HMIS (13/16, 81.3%) but only four (*n* = 16, 25.0%) did so in all countries of operation. DHIS2 (10/16, 62.5%) and Microsoft Excel (11/16, 68.8%) were the most common software platforms, and about a third (5/16, 31.2%) also used the local health authority HMIS system. Table 4 shows the different functional elements of HMIS employed by the participants. HMIS data were used for a diversity of activities – most frequently for pharmaceutical supply forecasting (14/16, 87.5%), reporting to donors (14/16, 87.5%), ongoing analysis of priority health needs (14/16, 87.5%), health service performance (13/16, 81.3%), and periodic quality reports (13/16, 81.3%). Conversely, feedback to beneficiaries (5/16, 31.3%) and cost-effectiveness analysis (3/16, 18.8%) were not common uses for HMIS. HMIS data were decreasingly available at higher levels of each organisation: at headquarters HMIS data was reported to be available by 64.9% (9/15) of key informants, but even at field office level they were not universally available (12/14, 85.7%).

**Table 4:** Number (%) of respondents using different elements of HMIS (*N* = 16)

Elements of HMIS	Yes N (%)	No N (%)	Unsure N (%)
Standardized menu of indicators and data elements	15 (93.8)	1 (6.2)	0 (0)
Aggregate service data	0 (0)	16 (100)	0 (0)
Electronic medical record	6 (37.5)	8 (50.0)	2 (12.5)
Pharmaceutical consumption data	15 (93.8)	1 (6.2)	0 (0)
Programme report generation	12 (75.0)	4 (25.0)	0 (0)
Data visualization and analysis	12 (75.0)	2 (12.5)	2 (12.5)
HMIS standard operating procedures	9 (56.3)	3 (18.7)	4 (25.0)
Internal training and technical user support	11 (68.8)	4 (25.0)	1 (6.2)

Approximately two-thirds of respondents (10/16, 62.5%) felt that HMIS was adequately funded and resourced in their organisation.

One participant commented that *“the organisation does not consider [it] a priority to engage a person to manage and oversee a MEAL* [monitoring, evaluation and learning] *strategy, therefore the workload falls on specific (thematic) technical advisers despite the dire need and the regular requests coming from bottom up”[P13].*

Another respondent noted limited capacity to make use of the data by saying *“a lot of data is collected but much more could be done to use it strategically and practically, if more resources and time were available (both in field and HQ).”[P6]* And a further comment highlighted the incentives for using and reporting on HMIS data: *“each country program is collecting their data in different forms based more on donors and cluster requirements rather than for quality improvement purpose[s]”[P15].*

However, the majority of participants (11/16, 68.8%) were able to cite positive examples of the use of HMIS data resulting in improved patient outcomes and advocacy for more resources. For example, one participant stated that *“analy[sis] of [the number] of trauma cases at primary health care level lead to [a] change of strategy regarding service delivery…leading in the end to [a] decrease in CFR* (case fatality rate) *for trauma cases in remote areas”[P2].*

#### Professional development

Although three quarters (12/16, 75.0%) of key informants said their organisation offered training and development packages for health sector staff, fewer than half (7/16, 43.8%) said their organisation had a protected budget for professional development, and even fewer identified a pathway to accredit staff (3/16, 18.8%). Just over half (9/16, 56.3%) reported a system in their organisation for appraising competencies and individual needs for continued professional development.

Regarding supervision of clinical and allied health staff, respondents reported that in most (9/16, 56.3%) or all (7/16, 43.8%) health care programmes that their organisation delivers, there is a designated lead for monitoring and supporting performance.

Free-text comments highlighted barriers to professional development, including lack or restricted use of funding, poor use of available training resources and the potential role of nepotism in accessing opportunities:

*“…there is a disparity between our field programmes, where training and development of health staff is a fundamental part of what we do… and HQ level where there is little to no funding or support for senior staff development…all programmes are donor funded…and the admin costs allocated to HQ support for programmes by donors do not extend to training and development of support staff”[P9].*

*“There is a budget proportion that is dedicated to training, but it is never fully utilized because the staff do not know that it exists and HR is usually not very open on sharing information about it”[P14].*

*“It is through personal relationships and personal endeavour that higher level staff get training opportunities”[P3].*

#### Health service audits

Health service audits were reported to occur by two-thirds of key informants (10/15, 66.7%) and half said audit was part of their organisational strategy or policies (8/16, 50.0%). Most key informants (8/13, 61.5%) were unsure how many healthcare audits were performed by their organisation during the last year, with the positive responses ranging from one to four. One respondent commented that *“I would hope many smaller clinical audits are done by staff in the field, we are trying to encourage this.” [P6].*

Several participants commented that audits were conducted, but this was “*not systematic*” [P5] with “*no formal structure*” for data collection or analysis [P13]. Another participant felt that audits were unfeasible: *“The larger audits we have done show this is quite resource-intensive, in terms of people and time. We usually focus on smaller problem-solving approaches, such as addressing issues involved in M&M* [morbidity and mortality] *reviews etc. Some plans are also made following medical error reports.”[P7].*

Among respondents whose organisation performed audits (*n* = 10), audit activities included: direct observation of patient care (10/10, 100%), pharmaceutical stock check (10/10, 100%), patient record review (9/9, 100%), pharmaceutical cold chain audit (9/10, 90.0%), health facility spot checks (9/10, 90%), health service data audit (8/10, 80.0%), procedural compliance checks (6/10, 60.0%), audit of staff training and competency (5/10, 50.0%) and audit of incident outcomes (5/10, 50.0%). Audit findings were mainly shared with staff involved in the audit (9/10, 90.0%), to a much lesser extent local health authorities (4/10, 40.0%) and beneficiaries (1/10, 10.0%), and never with response co-ordination mechanisms (0/10, 0.0%).

Further, the use of audits to improve service performance - rather than for monitoring compliance with financial or administrative regulations - was not always valued in surveyed organisations. One respondent noted that *“audits at point of health service delivery are done constantly as part of our programming, but at HQ/programme management level there are no standardised audit systems or templates to cover overall programme effectiveness.”[P9].*

#### Incident management

Just over half of key informants (10/16, 62.5%) responded that there was a system for reporting and investigating clinical incidents in their organisation, and eight (*n* = 16, 50.0%) indicated that incident reporting was mandatory. However, only six (*n* = 15, 40.0%) reported an established process for their investigation. Related guidelines were available in nine organisations (*n* = 16, 56.3%), and 10 (*n* = 16, 62.5%) gave staff and beneficiaries information on how to report incidents. Similarly, a standardized incident reporting form was available in nine (*n* = 15, 60%) organisations, and only three (*n* = 15, 20.0%) had a protected budget for incident management activities.

The fact that many organisations work predominantly within local health systems appears to create some division of responsibility for clinical incident reporting and investigation. This was cited by several respondents who felt it was outside of their organisation’s remit, mentioning that *“where clinical care is provided, there are local incident reporting systems in place”(P8)* and *“all reporting will go through [local Ministry of Health]”* or otherwise using *“partners protocols and the accountability team rather than the health team”(P3).*

Respondents highlighted underreporting [P6] and the perception that less serious incidents are not appropriately acted upon [P5]. Table 5 suggests limited dissemination of feedback about incidents outside of healthcare staff and highlights that between a quarter (3/14, 21.4%) and half (7/15, 46.7%) of key informants do not know with whom incident investigation findings are shared. One key informant [P9] described a recent positive change in practice from a system in which reports on incident investigations at field level were rarely fed upwards to headquarters to now having yearly reporting of incidents to the organisation board.

**Table 5:** Incident management – to whom are findings of incident investigations made available? (Variable N as stated)

To whom are findings of incident investigations made available?	Yes N (%)	No N (%)	Unsure N (%)	Total N
Health care staff	9 (60)	2 (13.3)	4 (26.7)	15
Donors	6 (42.9)	5 (35.7)	3 (21.4)	14
Partner organisations	5 (35.7)	5 (35.7)	4 (28.6)	14
Affected individuals	7 (46.7)	2 (13.3)	6 (40)	15
Affected communities	3 (20)	5 (33.33)	7 (46.7)	15

#### Evidence-based practice

We first asked about the use of information at the stage of designing a humanitarian health project. All respondents indicated using a wide range of information: defining the target population, understanding the current health and disease status of that population and the current level of demand for health programming, and identifying vulnerable and high-risk groups as systematic aspects of needs assessment. Cost and time effectiveness (9/15, 60.0%) and beneficiary representation (9/16, 56.3%) were less frequently considered.

Concerning monitoring the performance of ongoing projects, fewer than half (7/16, 43.8%) reported consistent monitoring of the impact on the population’s health, 10 (*n* = 16, 62.5%) always compared outcomes to pre-defined targets, two (*n* = 16, 12.5%) always compared outcomes to similar programmes within their organization and two (*n* = 16, 12.5%) always compared outcomes to similar programmes in other organizations. Just over half (9/16, 52.3%) always compared outcomes to international standards.

Five key informants reported that quality improvement is a routine component of their organisation’s health programmes (*n* = 15, 33.3%), and a further eight (*n* = 15, 53.3%) that QI is done on an ad-hoc basis. Two (*n* = 15, 13.3%) said QI did not feature in their health programmes at all. The most common barriers to QI were lack of time (9/16, 56.3%), knowledge (9/16, 56.3%) and funding (8/16, 50.0%). Five (*n* = 16, 31.3%) respondents also felt unsure that results of QI initiatives would be acted upon and three (*n* = 16, 18.8%) agreed that concerns about repercussions for exposing sub-optimal practice would act as a barrier. One respondent commented *“We are trying to change the ‘culture’ to be less of blame and more of learning but this is an uphill battle…” [P6].*

When asked about the use of best practice technical guidelines (e.g. WHO recommendations), key informants reported that these were available for clinical (15/16, 93.8%) and other technical activities (including public health and monitoring and evaluation) (13/16, 81.3%). However, training on the use of guidelines was inconsistent (11/16, 68.8%), and they were not always translated into the local language (3/16, 18.8%). There was a formal process for reviewing the guidelines in three-quarters of cases (11/16, 73.3%) and most audited adherence to them, either routinely (4/16, 3%) or on an ad-hoc basis (11/16, 68.8%).

Two-thirds of respondents indicated that their organisation conducted research (10/16, 62.5%). Only half of these said their organisation had an internal ethical review board (5/16, 31.3%). The latter may reflect the fact that *“research is 99% operational research” [P13]* and may not require ethical approval.

In their comments, participants highlighted a general lack of evidence for humanitarian health action as increasing their reliance on expert opinion and translation of practice directly from high-income countries. Lack of funding for and importance placed on research was also noted, with one respondent suggesting that *“…programming staff are assuming that the interventions are evidence based so [are] not overly keen to include research in the budgets. This is also true of donors not wanting to fund for baselines etc so we can assess quality.” [P3].*

#### Pharmaceutical services

Pharmaceutical services appeared closely monitored and standardized. Almost all key informants reported that: there was a project lead for pharmaceutical supply and management (15/16, 93.8%), with policies, guidelines or standard operating procedures for pharmaceutical procurement (14/16, 87.5%), storage of pharmaceutical supplies (14/16, 87.5%), supply chain management (14/16, 87.5%), drug donation (14/16, 87.5%), safe drug disposal (14/16, 87.5%), and stock management and inventory (13/16, 81.3%).

Pharmacovigilance and supervision of rational prescribing were less common, with about two-thirds reporting supervisory arrangements (10/15, 66.7%) and just over half reporting guidelines (9/16, 56.3%) for rational prescribing. Only six (*n* = 16, 37.5%) reported conducting structured pharmacovigilance, and according to written comments, this related mainly to specified drugs using an incident reporting system.

#### Beneficiary feedback

Several participants commented that beneficiary feedback was a routine part of all services and integral to the organisation’s philosophy. However, responses suggested that this is implemented inconsistently. Only two (*n* = 15, 13.3%) key informants said that 100% of health projects incorporate beneficiary feedback, with a median of 70% (range 10 to 100%) of health projects doing so. Guidelines to collect feedback were available according to just under half (7/16, 43.8%) of respondents. Protected budgets and time for collecting and acting on beneficiary feedback were available systematically in only two cases (*n* = 16, 12.5%) and sometimes in a further nine (*n* = 16, 56.3%). Six (*n* = 16, 37.5%) reported that there was never a member of staff within each project designated to manage feedback. Half (8/16, 50.0%) of key informants said their field staff get adequate training on collecting beneficiary feedback, and fewer (6/16, 37.5%) that they had adequate training to process this feedback. A minority (7/16, 43.8%) reported using a grading scale for categorizing serious grievances to ensure an effective and adequate response. Table 6 shows the modalities used by participants to collect feedback.

**Table 6:** Beneficiary feedback – modalities of data collection for beneficiary feedback (*N* = 16)

Modalities of feedback	Yes N (%)	No N (%)	Unsure N (%)
Face to face by caregiver	14 (87.5)	2 (12.5)	0 (0)
Text message (SMS)	6 (37.5)	8 (50)	2 (12.5)
Questionnaire at delivery point	15 (93.8)	1 (6.2)	0 (0)
Suggestion box	15 (93.8)	1 (6.2)	0 (0)
Focus groups	15 (93.8)	1 (6.2)	0 (0)
Community meetings	14 (87.5)	1 (6.2)	1 (6.2)

Two respondents mentioned the incentives for collecting feedback as coming from donors or other external pressures, and data not being well used:

*“Although we have always gathered some feedback as a matter of course, the universality of this is in the most part due to fairly recent donor requirements…I'm not sure everyone is really using the feedback we now receive effectively to improve quality of their programming, yet.” [P8]*

*“The only open interest from the organisation on beneficiary and staff feedback is re: sexual assault and fraud, otherwise…there is no accountability mechanism in place” [P13]*

### Perceived importance and effectiveness of governance domains

**Fig. 2:**
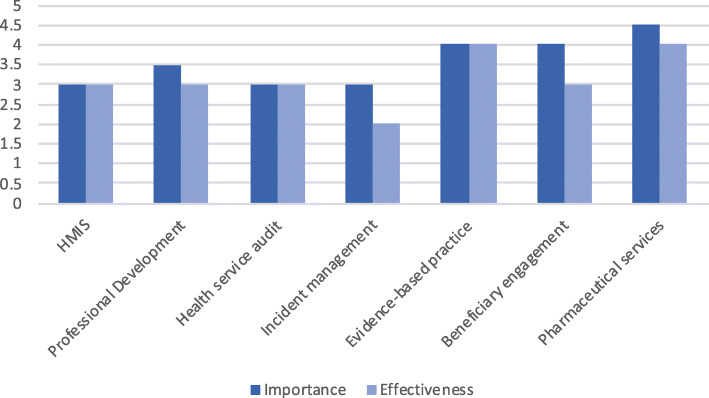
Median score rating for perceived importance and effectiveness of each governance domain (1-low, 5-high)/

For every governance domain, the distribution of responses on the perceived importance of the domain within the organisation was higher than for perceived effectiveness at tackling it (Fig. 2). Pharmaceutical services had the highest median score for importance at 4.5, and the lowest were HMIS, audit and incident management with a median score of 3. Pharmaceutical services and evidence-based practice had the highest score for effectiveness at 4, with incident management the lowest at 2. When all seven governance areas were combined, the median score for the importance of governance was 2 or ‘somewhat low’, and the median score for effectiveness was 1 or ‘low’.

## Discussion

To our knowledge, this is the first study to describe healthcare governance practice among large international humanitarian actors. Encouragingly, our results suggest that many are already implementing healthcare governance activities. However, these are inconsistently applied and generally not the expression of systematic policies and corresponding earmarked organisational resources: an ad hoc approach seems more common. Moreover, data and information on ‘problems’ (e.g. medical incidents) is infrequently available at headquarters level, where oversight and accountability should lie, and to beneficiary communities. Incentives such as donor requirements and external co-ordination mechanisms may drive governance activities, but this may not necessarily result in improved practice.

### Healthcare governance for both clinical and public health practice

The results of this survey mainly reflect clinical aspects of governance. This is despite our broader aim and definitions of governance and accountability (see methods) given in the first section of the questionnaire. As demonstrated by the paucity of published examples of healthcare governance in humanitarian aid, and their restriction to clinical activities, the concept of public health malpractice is relatively novel in the sector. While the use of the NHS framework in this study was intended to translate well-established concepts of clinical governance to public health, this design, as well as the technical background of the respondent, may have inadvertently prompted participants to report knowledge and experience of clinical governance rather than considering the broader definition we provided. This is a limitation of our study. These results may also reflect the fact that attempts to implement healthcare governance in humanitarian NGOs have similarly relied on direct translation of clinical governance practice from high-income healthcare settings, representing the genuinely narrow scope of healthcare governance policies in these organisations.

Despite the limitation described above, our study does provide some insights into governance practices related to public health. While there was some uniformity of practice in the use of public health information for programme design, there was substantial variability in its use for monitoring performance. This discrepancy perhaps reflects a lack of professional consensus or guidance on how the effectiveness of humanitarian health responses should be measured. Responses highlight the need for the more strategic and systematic use of public health data to inform decision-making and that current tools, such as audit, are underused for assessing public health programmes compared to clinical services.

Evidence-based practice – not only related to clinical but also public health programmes - was rated among the most effective areas. We can interpret this high rating in one of two ways: that proper use of quality information, guidelines and research is a genuine strength, or, an aspiration in contrast to the reality of poor collection and use of public health information, and the lack of good evidence for specific health interventions [37, 38].

### Cross-cutting findings

Our findings demonstrate potential for improvement across all domains of healthcare governance. Insufficient technical expertise and staff competence have repeatedly been identified as a contributor to humanitarian response failings [6, 20]. As illustrated by the quotes about professional development, our findings suggest institutional gaps that contribute to this problem, such as lack of assessment of staff competency and professional development needs, difficulty in accessing training, and lack of formal professional development opportunities. Good governance should enforce standards for recruitment, training and supervision of health staff. The WHO Emergency Medical Teams (EMT) programme [39] and the Humanitarian Quality Assurance Initiative [16] to accredit organisations against the Core Humanitarian Standard [15] are current attempts to certify actors based on agreed benchmarks of capability. There are disadvantages to these approaches, but at the system level, they may introduce accountability to accrediting bodies and incentivize self-improvement [4].

Engagement of beneficiaries was a recurrent weakness. Responses suggested poor commitment of resources and training for beneficiary engagement and concerns that feedback is not acted upon, and that this activity is often driven more by the need to satisfy donor requirements than to improve services. Furthermore, institutional culture seems to affect how such deficiencies can be countered. Participants mentioned fear of exposing malpractice and a culture of blame within organisations, challenges common to health systems with established governance cultures [40].

It is encouraging that technical guidelines were widely available to staff, and their use audited, if only to some extent. However, the lack of translation of these guidelines to local languages suggests that local staff not speaking UN languages may be excluded from accessing the guidance and expertise needed for effective decision-making. Such findings demonstrate how efforts to regulate humanitarian health action - healthcare governance being an example - may have unintended consequences [5] which should be considered in attempts at sector-wide reform.

### Limitations

Our findings may be subject to various biases. Firstly, selection bias may have arisen from the inclusion criteria. Our criteria privilege large organisations with a multi-country footprint, meaning our findings may not be representative of smaller, local entities, whose operational involvement in humanitarian action is ever-growing, or of donor institutions. Questions of governance are intrinsically linked to power through an emphasis on technocratic processes and expertise that are inevitably unevenly distributed in the humanitarian system [4]. We chose to study large international NGOs explicitly because of their influence on norms and reform in the humanitarian sector, but acknowledge this echoes the existing power structure rather than interrogating it. Further work to build consensus about humanitarian healthcare governance should examine the practices of other actors and consider the role of donors in incentivizing or distorting healthcare governance practice.

Equally, this study does not explore the elements of governance that relate to the relationship between large international NGOs and state health actors. This is a consideration distinct to healthcare governance in humanitarian settings and is likely to vary depending on the type and duration of the crisis and the relationship of the state to the affected population. Further study on where accountability lies in different crisis scenarios, particularly when NGOs are supporting rather than directly providing services, is needed.

Additional selection bias is likely as a result of survey attrition: while we do not believe that reticence to provide information explains the low survey response, actors who filled out the questionnaire may have more established headquarters technical expertise, and thus stronger governance provisions, than those who did not: as such, our findings may paint an overly optimistic picture.

Information biases are also plausible: our questionnaire was lengthy, potentially resulting in respondent fatigue and diminishing quality of responses (however, there was little attrition in responses across the questionnaire). Not all terms were defined, so respondents may have interpreted questions in ways specific to their organisation and their technical background, limiting the comparability of answers. Respondents (mainly senior headquarters experts) may not have detailed insight into all domains of the survey, or may have lacked perspective on how healthcare governance is implemented at lower levels of the organisation. Although participants were aware that their responses would not be published, we cannot discount that social desirability bias may have incentivized overly optimistic responses. Our questionnaire did not sufficiently distinguish between occasional and systematic practices (for example, respondents may have answered that their organisation operates a health information system even if, in fact, only some of its field projects do this, and irrespective of whether this is a centrally-driven or ad-hoc practice).

## Conclusion: towards a model of healthcare governance for humanitarian health responses

The humanitarian environment poses complex challenges for healthcare governance. Achieving consensus and consistency of practice is problematic as actors vary in size and technical expertise, individual NGOs may work in a range of crisis and sociocultural contexts and the degree to which they work with or separately to a state government and local health system varies. High turnover of program staff and short-term funding cycles – not to mention the moral urgency to respond as swiftly and decisively as possible - makes establishing robust, routinized processes for governance difficult. It was outside the scope of this study to explore all the contextual factors that influence healthcare governance in the humanitarian sector. However, it is clear that a unique model of healthcare governance, adapted for the challenges described above, is needed for humanitarian settings.

As a first step forward, consensus should be reached on what healthcare governance is and consists of in humanitarian response. In settings where public health needs evolve rapidly and exceptional threats (e.g. malnutrition, epidemics) arise, appropriate public health decision-making on what health services to offer, how and to whom becomes critical and is far more complex than in stable contexts where Ministries of Health take such decisions over long time horizons. We thus propose that ‘public health malpractice’ be considered within the scope of humanitarian healthcare governance, and better defined to help actors recognize and respond to it.

Conceptualizing humanitarian healthcare governance should also explicitly consider which actors and elements of the humanitarian system determine its effectiveness, and where relative power lies. For example, inadequate, conditional and short-term funding can obstruct needs-based decision-making and the ability to act on information about errors and poor performance. Competition over scarce resources may create perverse incentives not to document shortcomings that could be reputation-damaging [20]. Accordingly, the role and responsibility of donors in healthcare governance should be investigated.

Participant survey responses suggest a tension between organisational accountability and priorities and the need to support local health systems, whose standards may differ. Shared responsibility for service delivery may create confusion or an assumption that humanitarian actors should abdicate responsibility for what may ultimately be regarded as the remit of national health authorities. In response to recurrent failings of humanitarian aid in general, Spiegel proposes a model for co-ordination in humanitarian responses in which the intensity and autonomy of NGO and UN involvement varies based on different scenarios of local capacity and respect for humanitarian principles [41]. Such a model could also be applied to governance practices. Either way, consensus should be achieved on who is wholly or partly responsible for delivering adequate healthcare in different humanitarian response scenarios, or at the very least, a process for how this will be determined in different crisis contexts should be agreed .

Even if a sector-wide consensus on governance measures were reached, its monitoring and enforcement will remain problematic, as is the appropriate balance between punitive and supportive responses to malpractice. Consideration should be given to whether and what inter-agency body could take on this role. The World Health Organization is a natural candidate, given its technical pre-eminence and leadership of the humanitarian Health Cluster. However, the effectiveness and independence of the WHO in emergency response have come under scrutiny, particularly following the 2014–15 Ebola outbreak in West Africa [42, 43].

Ultimately, the perspective of patients and beneficiary communities needs to guide future directions: vulnerable, often disenfranchised populations affected by crises have as valid a claim to appropriate, effective healthcare as people in rich, stable countries. Accordingly, standards of humanitarian healthcare governance and accountability should not fall short.

## Supplementary Information

**Additional file 1.** Questionnaire.

## Data Availability

The data set used during this study are available from the corresponding author on reasonable request.

## Abbreviations

ALNAP: Active Learning Network for Accountability and Performance
CHS: Core Humanitarian Standard
EMT: Emergency Medical Teams
HAP-I: Humanitarian Accountability Partnership International
HMIS: Health Management Information Systems
HQAI: Humanitarian Quality Assurance Initiative
IASC: Inter-Agency Standing Committee
IFRC: International Federation of Red Cross and Red Crescent Societies
MEAL: Monitoring, Evaluation and Learning
MSF: Médecins Sans Frontières
NGO: Non-governmental organisation
NHS: National Health Service (UK)
OCHA: Office for the Co-ordination of Humanitarian Affairs
UN: United Nations
WASH: Water, Sanitation and Hygiene
WHO: World Health Organization
