# Supplementary material for: Healthcare governance during humanitarian responses: a survey of current practice among international humanitarian actors
Source: Confl Health. 2021 Apr 10;15:25. doi: 10.1186/s13031-021-00355-8 (PMC8035763; doi:10.1186/s13031-021-00355-8)
Supplement: Supplementary file 1 — Additional file 1. Questionnaire. [file 13031_2021_355_MOESM1_ESM.docx]

**Additional file 1 – Questionnaire**

# Introduction

Thank you for consenting to take part in this study. Please complete the relevant sections of the questionnaire to the best of your knowledge and provide examples where you feel relevant. Please provide all answers with reference to the organisation you are currently employed by. Some questions may prompt you to provide a piece of evidence or documentation, this is to help reduce the overall number of question in the study, despite this not being essential, your input would be greatly appreciated.

The questionnaire intends to analyse governance in a variety of areas of operation and practice. The governance components chosen in this study, aim to develop an understanding of governance theory and implementation within your organisation. These include: evidence based practice, auditing, incident investigation etc.

Throughout the questionnaire, ‘health’ implies any services provided under the health sector only (i.e. excluding nutrition, WASH and other sectors of humanitarian response).

In this study the following definition of governance and accountability has been utilised:

**Governance:** The set of rules, structures and mechanisms for collective decision-making employed by humanitarian health care actors and organisations, in the pursuit of providing effective and safe health care.

**Accountability:** The process by which humanitarian health care actors and organisations are taken into account for the decisions and actions they make in the delivery of health care by different stakeholders and those who they deliver care to.

The terms ‘direct’ and ‘support’ are also utilised in this study to describe the modality of service delivery. By direct the study refers to, the direct provision of a service by your organisation to an affected population. By support the study refers to, the provision of support to service providers (Ministries of Health, national or community NGO’s etc.) to ensure continuation and maintenance of critical and non-critical health services.

## Section 1: Details of respondent and organisation

To allow for some description of participating organisations and help us track survey response, please provide details of yourself and of your organisation. Note that the study report and any subsequent publication will not include any names or identifiers of survey respondents, and will not attribute specific data to any of the participating organisations. All identifiers will be removed from the database after the report is published.

1. What is your name? _______________________________________________
2. What is your job title? __________________________________________________
3. Where are you based (city, country)? _______________________________________
4. Name of the organisation on whose behalf you are providing information: __________________________________________
5. Which of the following health service areas does your organisation provide, and at which level of service delivery? Select all that apply – select ‘No’ if you do not support this health service at all.

| **Health Service Area** | **No – service not supported** | **Yes – at community-level or mass-campaign** | **Yes – at outpatient/primary level** | **Yes – at inpatient/secondary or tertiary level** |
| --- | --- | --- | --- | --- |
| Prevention and/or management of endemic infections  *(other than TB and HIV)* | Not supported | Direct  Support | Direct  Support | Direct  Support |
| Prevention and/or management of HIV/AIDS and/or tuberculosis | Not supported | Direct  Support | Direct  Support | Direct  Support |
| Prevention and/or management of epidemics | Not supported | Direct  Support | Direct  Support | Direct  Support |
| Sexual, reproductive, maternal and/or neonatal health services | Not supported | Direct | Direct  Support | Direct  Support |
| Prevention and/or management of non-communicable diseases | Not supported | Direct  Support | Direct  Support | Direct  Support |
| Mental health and psychosocial support services | Not supported | Direct  Support | Direct  Support | Direct  Support |
| Prevention and/or management of injuries | Not supported | Direct  Support | Direct  Support | Direct  Support |

1. Where does your organisation support services:

In multiple countries across more than one continent Yes  No  Unsure

In multiple countries within a single continent Yes  No  Unsure

In one country only Yes  No  Unsure

## Section 2: Overview of healthcare governance

This section of the questionnaire explores the general positioning of governance and accountability within your organisation.

1. Does your organisation have written policies or guidelines on internal governance and accountability?

Yes  No  Unsure

1. If yes, what sectors do these policies cover?

Health services  Generic humanitarian services  Unsure

1. Has the organisation set strategic, time-bound targets for governance and accountability?

Yes  No  Unsure

1. If yes, can you provide an example of a strategic document or policy where this is demonstrable? Either paste relevant text from a non-public document (please provide the title of the document), or provide a URL for a publicly available document we can consult.
2. Which of the following components of humanitarian healthcare governance has designated individuals and/or teams within the organisation that are responsible and accountable for their delivery?

Health Management Information System (HMIS) Yes  No  Unsure *Standardised system and associated resources for collection, management and analysis of data on health services supported by the organisation, for purposes of monitoring, evaluation and ongoing service improvement.*

Professional development of health sector staff Yes  No  Unsure  *Resources and policies whereby the organisation ensures that staff involved in the planning or delivery of health services it supports possess or acquire the competencies required for their role, have adequate supervision and regular appraisal of their performance.*

Health service audits: Yes  No  Unsure *Predictable arrangements for conducting and acting upon structured observations of the quality and adherence to standards of different health services supported or directly run by the organisation.*

Identification and management of clinical incidents: Yes  No  Unsure *Systems and processes for (i) rapidly detecting instances in which health services supported or directly run by the organisation may have endangered the safety and/or health of patients or other beneficiaries, and (ii) investigating, identifying root causes of and acting upon these instances.*

Evidence-based practice Yes  No  Unsure  *Systematic application of research evidence, contextual information, health service data and feedback from service providers and users to design, adapt and constantly improve health services supported by the organisation.*

Beneficiary feedback and engagement Yes  No  Unsure *The process and methodology by which beneficiaries of humanitarian health care programmes can provide information on their experiences through which service providers can monitor and evaluate practices to support service improvement.*

Pharmaceutical procurement and management Yes  No  Unsure *Systems, policies and organisational resources for timely procurement and management of quality, appropriate drugs and other pharmaceutical supplies.*

1. Which of the following governance components has an established escalation pathway to allow for concerns to be raised to the appropriate accountable level of the organisation?

Health service Audit Yes  No  Unsure

Clinical Incident management Yes  No  Unsure

Professional development Yes  No  Unsure

Pharmaceutical procurement and management Yes  No  Unsure

IT / HMIS Yes  No  Unsure Evidence-based practice Yes  No  Unsure Beneficiary feedback and engagement Yes  No  Unsure

1. Which of the following are utilised by your organisation?

Organisational risk register Yes  No  Unsure

A record of proactive efforts to identify, describe and rate current and past organisational risks, their likelihood, potential impact on services supported by the organisation (health or non-health) and agreed mitigation measures.

Regular quality reports Yes  No  Unsure

Periodic internal publication, at least on an annual basis, of reports presenting quantitative data on the quality of services (health or non-health) supported by the organisation, and. and identifying areas and recommendations for improvement.

Staff surveys or other feedback mechanisms Yes  No  Unsure

Representative surveys or other suitable feedback mechanisms (e.g. focus group discussions), carried out at least on an annual basis, and eliciting perceptions by the organisation’s staff of the appropriateness and performance of services (health or non-health) supported by the organisation (this could include one or more dimensions such as quality, efficiency, equity, managerial effectiveness, etc.)..

Publicly available board agenda and minutes Yes  No  Unsure

Publicly available (automatically or on request) minutes of the organisation’s board meetings, detailing issues discussed, processes for decision-making and key decisions -taken, in a transparent and clear way.

1. What sources of information does your organisation utilise for its health care programmes?

Routine programme data Yes  No  Unsure

Patient records Yes  No  Unsure

Incident reports Yes  No  Unsure

Morbidity and mortality meetings Yes  No  Unsure

Internal / external programme evaluations Yes  No  Unsure

Staff feedback Yes  No  Unsure

Beneficiary feedback Yes  No  Unsure

Audit findings Yes  No  Unsure

Health cluster reports Yes  No  Unsure

Patient safety reports Yes  No  Unsure

Case studies Yes  No  Unsure

## Section 3 – Specific governance components

In this section of the questionnaire we would like to explore in greater depth your organisation’s practices in regards to six key areas of healthcare governance. As above, please answer to the best of your knowledge.

We will also ask you to grade (on a scale from 1 to 5) the relative importance that you think your organisation places on each component, and your perception of how effective the organisation is in implementing the component. When grading, please try to be objective and keep in mind that responses will not be attributed to specific organisations in the study report.

Where space is provided, please feel free to supplement your answers with personal experience or recommendations, sharing best practices or perceived failings.

The questionnaire may also request a specific document or other pieces of evidence to support your answers. This is optional, but will help he study team to identify examples of good practice. Unless you explicitly indicate otherwise, we will assume that all documents are confidential to the organisation, and will thus not publish them or identify them in terms of titles and which organisations have developed or use them.

## Health Management Information System

1. Does your organisation employ a standardised Health Management Information System (HMIS)?

Yes – In all countries of operation  Yes – In some countries of operation  No  Unsure

1. If yes, which software platforms does the HMIS utilise?

DHIS – 1

DHIS – 2

Microsoft Excel

Microsoft Access

Whatever platform is used locally by the Ministry of Health or partner organisations

Other (please specify): ___________________________________________________

1. Of the following, which elements are part of your organisation’s HMIS:

Standardised menu of indicators and data elements Yes  No  Unsure

Collection of aggregate service data from health facilities Yes  No  Unsure

Electronic patient medical records Yes  No  Unsure

Pharmaceutical consumption data Yes  No  Unsure

Programme Report generation Yes  No  Unsure

Data visualisation and analysis Yes  No  Unsure

HMIS standard operating procedure Yes  No  Unsure

Internal training and technical user support Yes  No  Unsure

1. Approximately, what percentage of countries where your organisation currently supports humanitarian health services in, utilise a HMIS System?

________% Unsure

1. At what level of the organisation are HMIS data or HMIS generated reports immediately accessible?

Headquarters / Regional office Yes  No  Unsure

Country office Yes  No  Unsure

Field office Yes  No  Unsure

1. For which of the following indicators can you look up data from your desk for the second quarter of 2019, and for any country in which the organisation supports humanitarian health services?

Number of measles-containing vaccine doses administered Yes  No  n/a

Primary health care / outpatient services utilisation rate Yes  No  n/a

Proportional morbidity among children under 5y at primary / outpatient level

Yes  No  n/a

Inpatient case fatality ratio Yes  No  n/a

1. Does your organisation utilise HMIS data to provide feedback to staff on the performance of its services?

Yes  No  Unsure

1. In your opinion is the HMIS function within the organisation adequately funded and resourced?

Yes No

1. Which of the following are ways in which HMIS or otherwise sourced health service data are used by your organisation?

Scheduled reviews of health service performance by the board or senior management team

Yes  No  Unsure

Periodic (e.g. annual) quality reports Yes  No  Unsure

Pharmaceutical supply forecasting Yes  No  Unsure

Reporting to donors Yes  No  Unsure

Feedback to beneficiaries Yes  No  Unsure

Contribution to health cluster co-ordination Yes  No  Unsure

Operational research Yes  No  Unsure

Economic / cost-effectiveness analysis Yes  No  Unsure

Ongoing analysis of priority health needs Yes  No  Unsure

Ongoing analysis of health service performance Yes  No  Unsure

Other (please specify): _______________________________________________________

1. Using an example, could you outline an instance where health data collected through HMIS has positively affected health services supported by the organisation?

Yes  No  Unsure

1. On a scale of 1-5, what degree of importance is placed by your organisation on HMIS?

| **Importance** | | | | |
| --- | --- | --- | --- | --- |
| Low High | | | | |
| 1 | 2 | 3 | 4 | 5 |
|  |  |  |  |  |

(Please answer with an ‘x’ in the relevant box)

1. On a scale of 1-5, how effective is your organisation in its implementation of HMIS?

| **Effectiveness** | | | | |
| --- | --- | --- | --- | --- |
| Ineffective Very effective | | | | |
| 1 | 2 | 3 | 4 | 5 |
|  |  |  |  |  |

(Please answer with an ‘x’ in the relevant box)

1. Comments:

Please feel free to expand on any of the above points with personal or organisational experience or recommendations, share best practices or mention any challenges that exist within the scope of HMIS in humanitarian health care.

## Professional development of health sector staff

1. In your organisation is staff recruitment and promotion based on role specific technical competency frameworks?

Yes  No  Unsure

1. Does professional development of health sector staff have a protected budget within your organisation?

Yes  No  Unsure

1. Does your organisation offer any training and development packages to health sector staff in order to achieve role-specific competencies?

Yes  No  Unsure

1. To which types of health sector staff is role-specific training and development offered and what modalities are utilised (tick all that apply)?

|  | **HQ / Regional**  **Advisers** | **Emergency**  **Health staff** | **Country level directors** | **Field office managers** | **Clinical / Nursing supervisors** | **Frontline health staff** | **MoH / partner health staff** |
| --- | --- | --- | --- | --- | --- | --- | --- |
| Face to face internal courses |  |  |  |  |  |  |  |
| Face to face external courses |  |  |  |  |  |  |  |
| Distance learning |  |  |  |  |  |  |  |
| Mentoring / coaching |  |  |  |  |  |  |  |
| Refresher training |  |  |  |  |  |  |  |
| E-Learning / interactive guides |  |  |  |  |  |  |  |
| Internal publications / case studies |  |  |  |  |  |  |  |
| Podcasts / video |  |  |  |  |  |  |  |

1. Which of the following aspects of governance and accountability are offered as part of your organisations training and development manual and to whom (tick all that apply)?

|  | **HQ / Regional**  **Advisers** | **Emergency**  **Health staff** | **Country level directors** | **Field office managers** | **Clinical / Nursing supervisors** | **Frontline health staff** | **MoH / partner health staff** |
| --- | --- | --- | --- | --- | --- | --- | --- |
| Financial accountability of funds |  |  |  |  |  |  |  |
| Staff code of conduct / standards |  |  |  |  |  |  |  |
| Scope of practice |  |  |  |  |  |  |  |
| Health & Safety |  |  |  |  |  |  |  |
| Complaint handling |  |  |  |  |  |  |  |
| Humanitarian charter or Humanitarian principles |  |  |  |  |  |  |  |
| Equality and diversity |  |  |  |  |  |  |  |
| Audit training |  |  |  |  |  |  |  |

1. Is there a pathway whereby staff trained within your organisation can go on to receive a form of accredited certification?

Yes  No  Unsure

1. Is there an organisational system for assessing competencies of previously trained staff, and needs for continued professional development?

Yes  No  Unsure

1. Does the organisation have a staff performance appraisal system?

Yes  No  Unsure

1. Of the health care programme your organisation delivers, how many of these have a designated lead responsible for the supervision of staff (nurses, doctors, laboratory technician or pharmacist) to support and monitor performance?

Yes – in all health care programmes  Yes – in most health care programmes  No  Unsure

1. On a scale of 1-5, what degree of importance is placed by your organisation on training and development?

| **Importance** | | | | |
| --- | --- | --- | --- | --- |
| Low High | | | | |
| 1 | 2 | 3 | 4 | 5 |
|  |  |  |  |  |

(Please answer with an ‘x’ in the relevant box)

1. On a scale of 1-5, how effective is your organisation in its implementation of training and development?

| **Effectiveness** | | | | |
| --- | --- | --- | --- | --- |
| Ineffective Very effective | | | | |
| 1 | 2 | 3 | 4 | 5 |
|  |  |  |  |  |

(Please answer with an ‘x’ in the relevant box)

1. Comments:

Please feel free to expand on any of the above points with personal or organisational experience or recommendations, share best practices or mention any challenges that exist within the scope of training and development in humanitarian health care.

## Health Service Audits

1. Does your organisation perform any health service audits?

Yes  No  Unsure

1. How many health care audits were performed by your organisation during the last reporting year (calendar year or financial year, as per the organisation’s reporting schedule)?

____ (n) Unsure

1. Are health care audits a part your organisation’s strategy or policies, or otherwise part of the agreed ways of working of the organisation?

Yes  No  Unsure

1. Can you state what percentage of countries where you deliver humanitarian health projects benefited from at least one audit during the last reporting year (calendar year or financial year, as per the organisation’s reporting schedule)?

____ (%) Unsure

1. Which of the following activities are performed as part of your organisations auditing activities?

Direct observation of patient care delivery Yes  No  Unsure

Medical supply stock check Yes  No  Unsure

Pharmaceutical cold chain audit Yes  No  Unsure

Patient record / register review Yes  No  Unsure

Staff training and competency audit Yes  No  Unsure

Health service data audit Yes  No  Unsure

Health facility spot check: Yes  No  Unsure

Procedural compliance audit Yes  No  Unsure

Incident outcome audit Yes  No  Unsure

Other ______________________________

1. Of the following, with whom are audit findings shared with (select as many that apply):

Local health authorities

Staff involved in the audit

Beneficiaries (patients or community representatives)

Local coordination mechanisms (e.g. health cluster)

Other ­­­ ____________________

1. Please give a brief description of any pre-determined, systematic process by which the organisation puts in place remedial actions when problems are identified in an audit.

Unsure

1. In your opinion, on a scale of 1-5, what level of importance is placed on auditing by your organisation to adequately assess the quality of service delivery?

| **Importance** | | | | |
| --- | --- | --- | --- | --- |
| Inadequate Adequate | | | | |
| 1 | 2 | 3 | 4 | 5 |
|  |  |  |  |  |

(Please answer with an ‘x’ in the relevant box)

1. In your opinion, on a scale of 1-5, how effective is your organisation in implementation of its auditing process?

| **Effectiveness** | | | | |
| --- | --- | --- | --- | --- |
| Ineffective Very effective | | | | |
| 1 | 2 | 3 | 4 | 5 |
|  |  |  |  |  |

(Please answer with an ‘x’ in the relevant box)

1. Comments:

Please feel free to expand on any of the above points with personal or organisational experience or recommendations, share best practices or mention any challenges that exist within the scope of health service audits in your organisation.

## Identification and management of clinical incidents

1. Does your organisation have a system for detecting and reporting clinical incidents?

Yes  No  Unsure

1. Is reporting of clinical incidents mandatory within your organisation?

Yes  No  Unsure

1. How many clinical incidents were reported within your organisation during the last calendar year?

____ (n) Unsure

1. How many of these incidents have been investigated?

____ (n) Unsure

1. Does your organisation have an established process specifying the activities and timeline of a clinical incident investigation?

Yes  No  Unsure

1. Does your organisation have a specified frequency of meetings to review new incidents, ongoing investigations and the progress of remedial actions agreed for past incidents?

Yes  No  Unsure

1. What measures or ways of working, if any, are in place in your organisation to incentivise incident reporting, including by service providers who may be responsible for the incidents?

Unsure

1. Of the following, from which potential sources of information is your organisation able to learn about a clinical incident?

Beneficiary feedback/report (formal or informal) Yes  No  Unsure

Staff reporting Yes  No  Unsure

Regulator/inspection reports/audits Yes  No  Unsure

Analysis of routine health service data Yes  No  Unsure

Alert on counterfeit or sub-standard medical products issued by a manufacturer or other pharmaceutical supply actor Yes  No  Unsure

1. Which of the following are part of your organisation’s current practice on clinical incident management?

Guidelines on incident reporting Yes  No  Unsure

Information to staff and beneficiaries on how to report and incident

Yes  No  Unsure

A standardised incident report form Yes  No  Unsure

An incident severity rating scale/risk assessment tool to ascertain the level of investigation

required Yes  No  Unsure

Pre-defined incident escalation / decision-making pathways Yes  No  Unsure

Protected budget for investigation and reporting Yes  No  Unsure

1. Is your incident reporting system centralised, with data immediately available to designated accountable staff?

Yes  No  Unsure

1. To what extent are quality improvement projects part of your organisations health care programmes?

Routine part  Happens on ad hoc basis  Do not feature  Unsure

1. What are some of the barriers to performing quality improvement activities?

Not specifically part of anyone’s role

Lack of time 

Lack of knowledge about how to conduct them 

Lack of funding

Not sure that results would be acted upon

Concern about repercussions for exposing sub-optimal practice

Other: ________________________________________

1. To whom are findings of incident investigations made available?

Health care staff Yes  No  Unsure

Donors Yes  No  Unsure

Partner organisations Yes  No  Unsure

Affected individuals Yes  No  Unsure

Affected communities Yes  No  Unsure

1. Does your organisation have insurance or protected funds with which to litigate claims or compensate persons affected by a clinical incident?

Yes  No  Unsure

1. If an incident was a result of malpractice or negligence, does your organisation have policies and processes in place for appropriate sanctions and individual accountability?

Yes  No  Unsure

1. In your opinion, on a scale of 1-5, what degree of importance is placed by your organisation on detection and management of healthcare incidents?

| **Importance** | | | | |
| --- | --- | --- | --- | --- |
| Low High | | | | |
| 1 | 2 | 3 | 4 | 5 |
|  |  |  |  |  |

(Please answer with an ‘x’ in the relevant box)

1. In your opinion, on a scale of 1-5, how effective is your organisation in performing and reporting incident investigations?

| **Effectiveness** | | | | |
| --- | --- | --- | --- | --- |
| Ineffective Very effective | | | | |
| 1 | 2 | 3 | 4 | 5 |
|  |  |  |  |  |

(Please answer with an ‘x’ in the relevant box)

1. Comments:

Please feel free to expand on any of the above points with personal or organisational experience or recommendations, share best practices or mention any challenges that exist within the scope of incident reporting in humanitarian health care.

## Evidence-based practice

1. In what areas does your organisation use documented guidelines for practice in healthcare programs? Tick all that apply

Clinical

Technical (program design, M&E, reporting)

None

Unsure

1. What sources of information does your organisation utilise for the guidelines you use? Tick all that apply

Organisation’s own internal documents

Guidelines from another humanitarian healthcare organisation

International body (eg WHO, UNICEF)

National or regional guidelines from the country/ies where you provide services

National or regional guidelines from countries where you do not provide services  (neighbouring or developed countries)

1. Is training on the availability and use of role-specific guidelines a routine part of staff induction?

Yes  No  Unsure

1. Are guidelines translated into the language/s of the country where you are delivering services, such that local staff can use them?

Always  Sometimes  Rarely Unsure

1. Referring to internal guidelines, is there a formal process for reviewing them at regular intervals?

Yes  No  Unsure

1. Referring to clinical guidelines, in what form are they available to staff providing front-line services? Tick all that apply

Paper

Electronic

Not readily available in clinical environment

Unsure

1. Referring to clinical guidelines, is the adherence to guidelines audited?

Yes – Routinely  Yes – Ad hoc basis  No  Unsure

1. Does your organisation conduct research?

Yes  No  Unsure

1. Does your organisation have an internal ethical review system for scrutinizing and approving proposed research projects?

Yes  No  Unsure

1. Referring to internal guidelines, to what extent are the following sources of information considered when formulating them?

Programmatic data Always  Sometimes  Rarely Unsure

Audit data Always  Sometimes  Rarely Unsure

Incident reports Always  Sometimes  Rarely Unsure

Staff feedback Always  Sometimes  Rarely Unsure

Beneficiary feedback Always  Sometimes  Rarely Unsure

Other humanitarian healthcare agencies Always  Sometimes  Rarely Unsure

Expert opinion Always  Sometimes  Rarely Unsure

Published research Always  Sometimes  Rarely Unsure

International bodies (eg WHO, UNICEF) Always  Sometimes  Rarely Unsure

Other: ___________________________

1. Referring to internal guidelines, is a list of references used published within the guideline document?

Yes  No  Unsure

1. When performing a needs assessment for programme design, which one of the following components are mandatory within your organisations framework?

Definition of target population Yes  No  Unsure

Current health and disease status of target population Yes  No  Unsure

Projected health and disease status of target population Yes  No  Unsure

Identification of vulnerable/high risk populations Yes  No  Unsure

Health programme current demand Yes  No  Unsure

Health programme projected demand Yes  No  Unsure

Functionality of local health services Yes  No  Unsure

Local stakeholder analysis Yes  No  Unsure

Social and political context Yes  No  Unsure

Cost and time effectiveness Yes  No  Unsure

Beneficiary representation Yes  No  Unsure

1. How does your organisation measure the effectiveness of health care programs?

Monitoring of trends in health status (e.g. prevalence or incidence of disease, mortality rates, etc…) Always  Sometimes  Rarely Unsure

Compare outcomes to pre-defined program targets Always  Sometimes  Rarely Unsure

Compare outcomes to similar programs within the organisation (historic or ongoing in another setting) Always  Sometimes  Rarely Unsure

Compare outcomes to similar programs in other organisations (historic, same or different setting) Always  Sometimes  Rarely Unsure

Compare outcomes to international standards e.g. SPHERE

Always  Sometimes  Rarely Unsure

HMIS data Always  Sometimes  Rarely Unsure

1. On a scale of 1-5, what degree of importance is placed by your organisation on evidence-based practice?

| **Importance** | | | | |
| --- | --- | --- | --- | --- |
| Low High | | | | |
| 1 | 2 | 3 | 4 | 5 |
|  |  |  |  |  |

(Please answer with an ‘x’ in the relevant box)

1. In your opinion, on a scale of 1-5, operationally how effective is your organisation in its adherence to evidence-based practice?

| **Effectiveness** | | | | |
| --- | --- | --- | --- | --- |
| Ineffective Very effective | | | | |
| 1 | 2 | 3 | 4 | 5 |
|  |  |  |  |  |

(Please answer with an ‘x’ in the relevant box)

1. Comments:

Please feel free to expand on any of the above points with personal or organisational experience or recommendations, share best practices or mention any challenges that exist within the scope of evidence based practice in humanitarian health care.

## Beneficiary feedback and engagement

1. As a rough percentage, how many of your organisations health projects do you think incorporate some form of beneficiary feedback mechanism?

______________________________%

1. Do you have organisational guidelines in place to help design and conduct beneficiary feedback of the health services you deliver?

Yes  No  Unsure

1. Are health care projects assigned protected budgets and time to manage feedback activities?

Always  Sometimes  Rarely Unsure

1. At what level is field level feedback data reported?

Country Yes  No  Unsure

Regional Yes  No  Unsure

Headquarters Yes  No  Unsure

1. How often is beneficiary feedback reported alongside health project M&E data?

Always  Sometimes  Rarely Unsure

1. Are country / field staff adequately trained to:

Receive beneficiary feedback from a variety of sources Yes  No  Unsure

Process feedback in a standardised manner Yes  No  Unsure

1. Does each health project have a competent designated member or staffs responsible for analysing, amalgamating and reporting feedback data?

Always  Sometimes  Rarely Unsure

1. What modalities are utilised by your organisation to collect beneficiary feedback?

Face to face by caregiver Yes  No  Unsure

SMS Yes  No  Unsure

Questionnaire at delivery point Yes  No  Unsure

Suggestion box Yes  No  Unsure

Focus groups Yes  No  Unsure

Community meetings Yes  No  Unsure

1. In your opinion, how aware are beneficiaries of the purpose and process of feedback mechanisms in the health projects delivered by your organisation?

Always  Sometimes  Rarely Unsure

1. Which of the following beneficiary mechanisms does your organisation use to measure programme outcomes?

Focus group discussions Yes  No  Unsure

Health service patient surveys Yes  No  Unsure

Interviews from a sample of beneficiaries Yes  No  Unsure

Beneficiary complaints / concerns Yes  No  Unsure

1. When a serious grievance is raised, does your organisation have grading system to ensure effective and adequate response is given to the complaint?

Yes  No  Unsure

1. Does your organisation have a formalised mechanism for staff feedback and suggestions for project delivery improvements?

Yes  No  Unsure

1. In your opinion, on a scale of 1-5, what degree of importance is placed by your organisation on beneficiary feedback?

| **Importance** | | | | |
| --- | --- | --- | --- | --- |
| Low High | | | | |
| 1 | 2 | 3 | 4 | 5 |
|  |  |  |  |  |

(Please answer with an ‘x’ in the relevant box)

1. In your opinion, on a scale of 1-5, how effective is your organisation in its implementation to meet beneficiary feedback needs?

| **Effectiveness** | | | | |
| --- | --- | --- | --- | --- |
| Unsatisfactory Satisfactory | | | | |
| 1 | 2 | 3 | 4 | 5 |
|  |  |  |  |  |

(Please answer with an ‘x’ in the relevant box)

1. Comments:

Please feel free to expand on any of the above points with personal or organisational experience or recommendations, share best practices or mention any challenges that exist within the scope of evidence based practice in humanitarian health care.

## Pharmaceutical procurement and management

1. Which resources from the list below are available within your organisation for its pharmaceutical procurement and management?

Pre-qualification / approval of suppliers Yes  No  Unsure

Electronic stock management system Yes  No  Unsure

Centralised pharmaceutical advisory team Yes  No  Unsure

Standardised list of medical / non-medical supplies Yes  No  Unsure

Emergency stock supply management (Electronic / Physical) Yes  No  Unsure

1. Are there policies, guidelines or standard operating procedures in place for the following:

Pharmaceutical procurement Yes  No  Unsure

Stock management and inventory Yes  No  Unsure

Storage of pharmaceutical supplies Yes  No  Unsure

Drug quality assessment Yes  No  Unsure

Pharmaceutical supply chain management Yes  No  Unsure

Rational prescribing Yes  No  Unsure

Drug donation policy Yes  No  Unsure

Expired / damaged safe drug disposal Yes  No  Unsure

1. In any given project, can an individual be identified as the lead for pharmaceutical supply and management?

Yes  No  Unsure

1. At what levels within your organisation is stock out of essential and tracer drugs immediately available?

Headquarters / regional office Yes  No  Unsure

Country office Yes  No  Unsure

Field office Yes  No  Unsure

Health facility Yes  No  Unsure

1. Does your organisation have auditory / supervisory arrangements to ensure rational drug prescribing?

Yes  No  Unsure

1. Does your organisation carry out structured pharmacovigilance?

Yes  No  Unsure

1. If yes, can you provide a brief description or documentation of how this operates?
2. In your opinion, on a scale of 1-5, what degree of importance is placed by your organisation on pharmaceutical procurement and management chain?

| **Importance** | | | | |
| --- | --- | --- | --- | --- |
| Low High | | | | |
| 1 | 2 | 3 | 4 | 5 |
|  |  |  |  |  |

(Please answer with an ‘x’ in the relevant box)

1. In your opinion, on a scale of 1-5, how effective is your organisation in its implementation to meet health programme pharmaceutical procurement and management needs?

| **Effectiveness** | | | | |
| --- | --- | --- | --- | --- |
| Ineffective Very effective | | | | |
| 1 | 2 | 3 | 4 | 5 |
|  |  |  |  |  |

(Please answer with an ‘x’ in the relevant box)

1. Comments:

Please feel free to expand on any of the above points with personal or organisational experience or recommendations, share best practices or mention any challenges that exist within the scope of pharmaceutical procurement and management in humanitarian health care.

## Concluding remarks:

1. Please feel free to provide any additional information you feel is pertinent to the study from the perspective of yourself and your organisation or a more generalised commentary on governance and accountability of the system overall.

Thank you for your participation and time, your contribution is greatly appreciated in this study. Please feel to get in touch if you have any further questions and upon completion of the study, the findings and a report will be shared with you.
